# Prevention of Bacterial Biofilm Formation on Soft Contact Lenses Using Natural Compounds

**DOI:** 10.1186/s12348-017-0129-0

**Published:** 2017-04-18

**Authors:** Amira M. El-Ganiny, Ghada H. Shaker, Abeer A. Aboelazm, Heba A. El-Dash

**Affiliations:** 1 Microbiology and Immunology Department, Faculty of Pharmacy, Zagazig University, Zagazig, Egypt; 2 Microbiology and Immunology Department, Faculty of Medicine, Benha University, Benha, Egypt

**Keywords:** Biofilm, Contact lenses, *Calendula officinalis*, *Buddleja salviifolia*

## Abstract

**Purpose:**

In eye care field, contact lenses (CL) have a great impact on improving vision, but their use can be limited by ocular infection. CL- associated infections can be reduced by good attention to CL storage case practice. CL-care solutions should be able to control microbial growth on CL.

The aim of the study was to evaluate and compare the efficacy of CL-care solutions (found in Egyptian market) with some natural compounds in removal and inhibition of bacterial biofilm formed on soft CL.

Clinical isolates were recovered from patients having conjunctivitis from Benha University Hospital and identified microbiologically. Quantification of biofilm was done using microtiter plate assay. Three multipurpose CL-care solutions were examined for their ability to remove and inhibit biofilm. Also four natural extracts having antibacterial activity and are safe on eye were tested for their anti-biofilm activity.

**Results:**

The major bacterial isolates from eye infections were *Pseudomonas aeruginosa* (36%) and *Staphylococcus spp.* (37.8%). Only 33.3% of isolates showed ability to produce weak to moderate biofilm. The tested multi-purpose CL-care solutions showed moderate ability to remove preformed biofilm. Among the tested natural compounds, *Calendula officinalis* and *Buddleja salviifolia* extracts showed an excellent efficacy in inhibition of biofilm and also removal of preformed biofilm.

**Conclusion:**

This study demonstrated that isolates from infected eye and CL-cases showed weak to moderate biofilm formation. *Calendula officinalis* and *Buddleja salviifolia* extracts showed excellent effect on inhibition and removal of biofilm, these extracts could be added into CL-care solutions which could markedly reduce eye-infections during CL-wear.

## Introduction

The human's eye is protected by many factors that prevent ocular infections. However, in certain circumstances, microorganisms gain access to the eye causing variety of infections*.* The most common bacteria that can cause eye infections are *Staphylococcus aureus*, *Sterptococcus pneumoniae*, *Pseudomonas aeruginosa*, *Hemophilus influenza* and *Klebsiella species* [1].

In the eye care field, contact lenses (CL) have a great impact on improving vision, but their use can be limited by ocular infection. CL-wear is the most important risk factor for microbial infections. Wearers of soft lenses are at higher risk than other types of lenses [2]. Wearing contact lenses is associated with changes in the ocular microbiota, the microbiota of ocular conjunctiva was found to be similar to that of skin under the eye [3]. Gram-negative bacteria are the predominant cause of CL-related microbial keratitis with *Pseudomonas spp.* being the most commonly isolated organism [4–7], while *Staphylococcus spp.* and *Serratia spp.* come next [8, 9]. Infections are more likely if there is poor lens hygiene [10]. Microbial infections associated with use of CL may be considerably reduced by attention to risk factors related to CL storage case practice [11, 12]. It is essential that CL-care solutions should be able to sufficiently decrease the amount of pathogens in order to decrease the risk of CL-related infections [13]. In addition, some infectious ocular diseases are due to bacterial biofilm formation, biofilm is highly resistant to many antimicrobials [14, 15]. Hence, CL-care solutions should have ability to reduce or prevent biofilm formation on CL.

For a long time many people around the world have used plants for therapy. Recently the number of people using plant extracts for therapy increased and the numbers remain on the rise [16, 17]. Many natural compounds have been used to kill infectious pathogens, others were used for eye remedies because they are known to be safe on eye [18, 19]. For example, honey was used for treatment of CL induced corneal ulcer; and it shows high *in vitro* antibacterial activity against ocular isolates [20]. Moreover, honey exhibits anti-biofilm and anti-inflammatory properties, and thus becomes an interesting ophthalmologic agent [21]. The flowers of *Calendula officinalis* have a good antimicrobial and anti-biofilm activities [22, 23]*.* And owing to their anti-inflammatory and healing properties, *C. officinalis* extracts are applied externally to treat conjunctivitis [24]. *Jasminum* flowers has antibacterial and antifungal activities due to its essential oil content, also leaf extract of *Jasminum* was used in treatment in of inflamed eyes [25, 26]. *Buddleja salviifolia* leaves were used for treatment of eye infections by tribes in South Africa. The extract of *Buddleja officinalis* leaves was used for partial treatment for surface diseases of eyes, it also exhibited a broad spectrum antibacterial activity [27, 28].

The need to prevent, reduce, or eliminate microbial biofilm is becoming an important constraint. Strategies, such as coatings with anti-biofilm and developing anti-biofilm therapeutics, are promising avenues to reduce the risk of biofilm-associated ocular infection [29]. In the current study honey, jasmine oil, *Calendula Officinalis* petal extract and *Buddleja salviifolia* leaves extract were assessed for their activity on inhibition and removal of bacterial biofilm on microtiter plates and soft contact lenses in comparison to three multi-purpose CL-care solutions found in Egyptian market; Renu, Opti-free and Perfect care solutions.

## Results

### Identification of bacterial isolates

A total 111 isolates were recovered from 184 specimens, 81 isolates from clinical samples and 30 isolates from CL cases. The isolated organisms were identified using standard microbiological tests. Among the 111 isolates, 41 (36.9%) were Gram positive*.* including 22 (19.8%) *S. aureus,* 10 (9%) *S. epidermidis* and 9 (8.1%) *S. saprophyticus*. The seventy Gram negative isolates include, 40 (36%) *P. aeruginosa,* 9 (8.1%) *K. pneumonia,* 7 (6.3%) *Serratia spp.,* 6 (5.4%) *Moraxella catarralis,* 6 (5.4%) *M. lacunata,* and only 2 (1.8%) *E. coli* (Table 1).

**Table 1:** The distribution of specimens and type of bacterial isolates

Specimens/MO	Infected eye	Lens case	Total	
	No.	No.	No.	%
No of Specimens	116	68	184	100
Negative Specimens	49	46	95	51.6
Positive specimens	67	22	89	48.4
Multi-infection specimens	14	8	22	
Uni-infection specimens	53	14	67	
*S. aureus*	20	2	22	19.8
*S. epidermidis*	8	2	10	9
*S. saprophyticus*	2	7	9	8.1
*P. aeruginosa*	30	10	40	36
*Klebsiella spp.*	9	0	9	8.1
*Serratia spp.*	0	7	7	6.3
*M. lacunata*	6	0	6	5.4
*M. catarrhalis*	6	0	6	5.4
*E. coli*	0	2	2	1.8
Total isolates	81	30	111	100

### Assessment of biofilm formation by spectrophotometric method

All isolates were tested for biofilm production. Only 12 isolates (10.8%) were moderate biofilm forming, 25 isolates (22.5%) were weak biofilm forming and 74 (66.6%) were non biofilm forming. Twenty two *P. aeruginosa* and eight *S. aureus* isolates were biofilm forming. Only four *S. epidermidis*, two *S. saprophyticus* and one *K. pneumonia* isolates were weak biofilm producers (Table 2).

**Table 2:** Distribution of biofilm forming isolates

Microorganism	Moderate biofilm	Weak biofilm	Total
*P. aeruginosa*	10	12	22
*S. aureus*	2	6	8
*S. epidermidis*	0	4	4
*S. saprophyticus*	0	2	2
*Klebsiella spp*	0	1	1

### Effectiveness of disinfectant solutions and natural compounds on inhibition of biofilm

The MICs for the tested disinfectant solutions and natural compounds were determined by broth micro-dilution method against all biofilm forming isolates. The MIC_90_ for both Renu and Opti-free were 0.125 of their original concentration. While MIC_90_ of Perfect solution was 0.25 of its original concentration. The MIC_90_ for Honey, *Calendula officinalis*, Jasmine oil and *Buddleja salviifolia* extract were 125 μL/mL, 31.2 μL/mL, 15.6 μL/mL. 31.2 μL/mL, respectively.

**Fig. 1:**
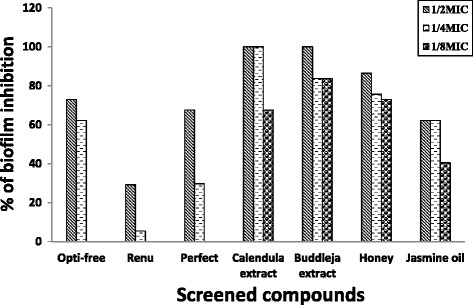
Percentage of biofilm inhibition by screened CL-care solutions and natural compounds

Sub-MICs (½ and ¼ MIC) of disinfectants solutions were tested for biofilm inhibition capacity. In case of ½ MIC, Opti-free was able to prevent 72.97% of tested isolates from biofilm formation. Perfect solution prevents 67.56% of tested isolates, while Renu was able to prevent only 29.72% of tested isolates (Fig. 1).

For natural compounds, $\frac{1}{2},\kern0.5em \frac{1}{4}$, and $\frac{1}{8}$ MIC of Honey were able to inhibit 86.5%, 75.7%, and 73% of tested isolates from biofilm production respectively. Also, $\frac{1}{2},\kern0.5em \frac{1}{4}$, and $\frac{1}{8}$ MIC of *Calendula* extract were able inhibit 100%, 100%, and 67.6% of tested isolates from biofilm production respectively. For *Buddleja salviifolia* extract, $\frac{1}{2},\kern0.5em \frac{1}{4}$, and $\frac{1}{8}$ MIC were able to inhibit 100%, 83.8%, and 83.8% of tested isolates from biofilm production, respectively. Finally, $\frac{1}{2},\kern0.5em \frac{1}{4}$, and $\frac{1}{8}$ MIC of Jasmine oil were able to inhibit 62.2%, 62.2%, and 40.5% of tested isolates from biofilm formation, respectively (Fig. 1).

### Effectiveness of disinfectant solutions and natural compounds on removal of pre-formed biofilm

**Fig. 2:**
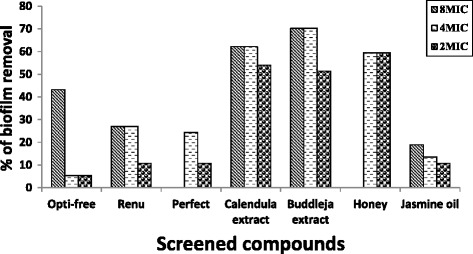
Percentage of biofilm removal by screened -care solutions and natural compounds

Different concentrations (8, 4, 2 fold MIC) of disinfectant solutions were tested for their biofilm removal effect. All CL-care solution showed low effect on removal of preformed biofilm. Eight fold MIC of Opti free solution is considered the best in removal of biofilm as it removed biofilm formed by 43.2% of isolates. While eight fold MIC of Renu solution was able to remove biofilm formed by 27% of tested isolates and reduced strength of biofilm from moderate to weak in 21.6% of isolates. Four fold MIC of both Perfect and Renu solutions were able to remove preformed biofilm by 24.3% of tested isolates (Fig. 2).

The biofilm formed by tested organisms were also exposed to 8, 4 and 2 fold MIC of Jasmine oil, *Calendula officinalis* extract, and *Buddleja salviifolia* extract and to 4 and 2 fold MIC of Honey. For Honey 4 and 2 fold MIC were able to remove biofilm formed by 59.5% for both concentrations. For *Calendula officinalis*, 8, 4 and 2 fold MIC were able to remove biofilm formed by 62.2%, 62.2% and 54.1% respectively. Regarding *Buddleja salviifolia,* 8, 4 and 2 fold MIC were able to remove biofilm formed by 70.3%, 70.3% and 51.4%, respectively. Jasmine oil showed the least effect, 8, 4 and 2 MIC were able to remove biofilm formed by 18.9%, 13.5% and 10.8% of isolates, respectively (Fig. 2).

### Effectiveness of *Calendula* and *Buddleja* extracts on inhibition of biofilm formation and removal of preformed biofilm on CL

For both *Calendula* extract and *Buddleja* extract, ½ and ¼ MIC were tested on biofilm forming *S. aureus* and *P. aeruginosa* isolates. For *Calendula*, ½ MIC was able to inhibit biofilm formation on CL for the two isolates but ¼ MIC inhibits *P. aeruginosa* and reduces the ability of *S. aureus* to form biofilm on CL. While ½ MIC and ¼ MIC of *Buddleja* were able to inhibit biofilm formation for the two isolates.

For both *Calendula* and *Buddleja* extracts, 4 and 8 fold of MIC were tested on biofilm forming *S. aureus* isolate and *P. aeruginosa* isolates. Four and eight MIC of *Calendula* were able to remove preformed biofilm on soft CL. Four and eight MIC of *Buddleja salviifolia* were able to remove preformed biofilm of *S. aureus* but it only weakens the biofilm formed by *P. aeruginosa*.

## Discussion

The eye is protected by a number of natural defence mechanisms that reduce the eye infections. However many ocular infections are caused by the use of soft CL [30]. The current study aims to screen for the biofilm forming isolates from infected eyes and CL-cases and to evaluate the anti-biofilm activity of some natural compounds in comparison to three CL-care solutions.

In the present study *P. aeruginosa* was the most isolated organism from eye infection. It was recovered at a frequency of 37%, followed by *S. aureus* (24.7%) and *Klebsiella spp.* (11%). Previous studies recovered *P. aeruginosa* at lower frequencies ranging from 1.25 - 19% [1, 30, 31]. The percentage of *S. aureus* was slightly lower than the 32.3% reported previously [32], but quite similar to previous studies that recovered *S. aureus* at frequency of 22%, and 23.6% [31, 33].

The major isolates from CL-cases were *P. aeruginosa, Serratia spp. and S. saprophyticus. P. aeruginosa* was isolated in the highest frequency (33.3%) which agree with the results of previous reports [34, 35]. In current study *Serratia* was recovered only from CL-cases in a frequency of 23.3% which is also near to what reported previously [34, 35].

In this study, only *P. aeruginosa, Staphylococcus spp and klebsiella spp* were able to form weak to moderate biofilms. 55% of isolated *P. aeruginosa* were biofilm producer. A previous study reported lower percentage (33%) [36], while Oncel and his colleagues found similar result, where 60% of isolated *P. aeruginosa* formed biofilm [37]. In this study, 36.4% of isolated *S. aureus* were biofilm producer, which is lower than the 51.9% reported previously [30].

The biofilm forming isolates were exposed to sub-MIC concentrations of the three disinfectant solutions found in the Egyptian market to test their ability to inhibit biofilm formation. Our results showed that the three disinfectant solutions have moderate activity against biofilm removal without the rubbing step that is recommended by the manufacturer but some consumers did not comply with lens hygiene procedures [38]. It worth mentioning that the current study is the first to assess anti-biofilm properties of CL-care solutions found in the Egyptian market against bacterial biofilms grown on both polystyrene microtiter plates and soft CL.

Previous study reported that all the tested lens care solutions were effective against planktonic bacterial growth, and were ineffective against bacterial biofilm *in vitro* [39]. The ability of Renu and Opti-free solutions to remove biofilm formed on silicon hydrogel lenses was assessed previously, the results were unsatisfactory when steps of rubbing and rinsing of lenses were omitted [38].

The tested organisms were also exposed to sub-MIC concentrations of four natural compounds to evaluate their ability to inhibit and remove biofilm. *Buddleja salviifolia* and *Calendula officinalis* extracts showed the highest activity in inhibiting biofilm formation followed by honey then jasmine oil. Also *Buddleja salviifolia* and *Calendula officinalis* extracts have excellent effect on removal of preformed biofilm, while honey has moderate effect. It was reported previously that honey was able to penetrate biofilm formed *K. pneumoniae* and *P. aeruginosa* [40]. However in our study honey showed moderate activity in removal of biofilm when compared with other tested compounds. Although Jasmine oil showed low MIC (15.6 μL/mL) in our study, but it cannot be considered effective in removal of preformed biofilm.

The phytochemical composition, biological activity and safety of *Calendula officinalis* extracts are well documented [41–43]. The main active constituents in *Calendula* are terpenoids, flavonoids, coumarines, quinones and volatile oils [42]. It was previously reported that the extracts of *Calendula officinalis* decreased the adherence of bacteria on glass tubes, inhibited adhesion on polystyrene surface and caused biofilm detachment [24]. Regarding safety issue, acute toxicity studies in rats and mice indicate that the extract is relatively nontoxic. Minimal ocular irritation was seen with one formulation containing lipophilic extract of *C officinal* and no irritation with other extracts [43, 44].

*Buddleja officinalis* was used by Chinese Medicine and elsewhere to treat eye diseases, with flavonoids as its effective part [27]. Phytochemical analysis of *Buddleja* extracts identified polyphenols, flavonoids and phenylethanoid as major components [45, 46]. *Buddleja* showed antimicrobial activity against *Bacillus subtilis, S. aureus, E. coli* and *K. pneumoniae* supporting the traditional use of the plant in the treatment of eye infections [28]. *In vivo* studies in animal models showed that eye drops containing *Buddleja* can be used safely to treat dry eye [27, 47].

## Conclusions

In conclusion, the previous studies supported the results of current study that tested natural compounds could be used as prophylactic agent to prevent ocular infections especially those caused by CL-wear. To our knowledge, the current study is the first to assess the anti-biofilm activity of both *Buddleja salviifolia* and Jasmine oil. Also it is the first description of a model that study the anti-biofilm activity of both *Calendula officinalis* extract and *Buddleja salviifolia* extract on soft CL. Our study showed that *Calendula officinalis* and *Buddleja salviifolia* extracts have excellent effect on inhibition of biofilm formation and removal of preformed biofilm which make them promising agents that can be added to new more effective CL-care solutions.

## Material and methods

### Bacterial isolation and identification

A total number of 184 specimens were collected, 116 samples were obtained from Ophthalmology Department in Benha Educational University Hospital, Benha, Egypt. While 68 specimens were collected from CL-cases of lenses users. The study was approved by the Ethical Committee of our university.

All specimens were taken by moistened sterile swab. The swabs were cultured on chocolate agar, blood agar and MacConkey agar, then incubated at 37 °C for 24 hours. Gram stained films were prepared from the isolated colonies*.* Standard Microbiological tests were also done to identify each isolate to the species level [48].

### Screened compounds

Three soft CL-care solutions available in Egyptian market were tested: Renu Multi-purpose solution (Bausch and Lomb-IOM, Milan, Italy), Opti-free solution (Alcon Laboratories, Inc. Fort Worth, Texas, USA) and Perfect care protein remover (Orchidia Pharmaceutical Industries, El Obour, Egypt). Four natural compounds were also tested including honey, jasmine oil, leaves extract of *Buddleja salviifolia* and petal extract of *Calendula officinalis*. Honey was purchased from Isis Company (Cairo, Egypt), Jasmine oil, *Buddleja salviifolia* leaves extract and *Calendula officinalis* flowers extract were prepared by Morgan chemicals company (Cairo, Egypt), upon authors request. Jasmine oil was extracted from *Jasminum officinale* petals by hydro-distillation [49] The powdered *Calendula officinalis* flowers were subjected to extraction with ethanol using soxhlet apparatus as described previously [50] The powdered leaves of *Buddleja salviifolia* were extracted with 20% aqueous methanol as reported previously [28].

### Detection of biofilm forming isolates

All isolates were screened for their ability to form biofilm by microtiter plate method with some modifications [51]. Overnight cultures of isolates from trypticase soya agar (TSA) plates were inoculated in tryptone soya broth (TSB), and the turbidity was adjusted to 0.5 McFarland. The suspension were further diluted 1:100 to obtain density of 10^5^-10^6^ cells/mL. Aliquots of 100 μL were distributed in 96-well microtiter plate containing 100 μL of TSB with 2% glucose (TSB-glu), negative control wells were included. The plates were incubated for 48 hours at 37 °C. The content of each tube was aspirated and then washed three times with phosphate buffered saline (PBS) to remove any non-adherent bacteria. 200 μL of 99% methanol was added to each well for 15 min to fix biofilm. The wells were decanted, left to dry, and stained with 200 μL of 2% Hucker Crystal Violet (CV) for another 15 min. Excess stain was rinsed off gently by water. The plates were air dried; the bound dye was solubilized with 200 μL of 33% (v/v) glacial acetic acid. The Optical Density (OD) was measured at 570 nm using spectrophotometer (UV-1800 Shimadzu, Japan). The test was made in triplicates. Based on the measured ODs, the tested isolates were classified into four categories; non-adherent, weakly adherent, moderately adherent, and strongly adherent [51].

### Determination of minimum inhibitory concentration (MIC) of Screened compounds

The MICs of disinfectants (CL-care solutions and natural compounds) were determined by broth dilution method according to CLSI [52]. Colonies from biofilm forming isolates were touched with a sterile loop and transferred to Muller-Hinton broth (MHB), the turbidity was adjusted to 0.5 McFarland. The suspension was then diluted 1:100 in MHB.

Two fold serial dilutions of each disinfectant agent were prepared using microtiter plates, 100 μL of each dilution are placed in adjacent wells. 100 *μ*L of prepared inoculum were added to each dilution, control wells were included. Plates were incubated at 37 °C for 18-20 hours. The MIC was taken as the lowest concentration of disinfectant which inhibits bacterial growth.

### Effectiveness of screened compounds on inhibition of biofilm formation

Overnight cultures of isolates from TSA plates were inoculated in TSB and the turbidity was adjusted to 0.5 McFarland which was further diluted 1:100 to obtain density of 10^5^-10^6^ cells/mL. Aliquots of 100 μL were distributed in 96-well microtiter plate containing 100 μL of sub-MIC of tested disinfectants (1/2 and 1/4 and 1/8 MIC) and incubated in stationary conditions for 48 hours at 37 °C. The content of each well was aspirated, washed three times with PBS, fixed with 200 μL of 99% methanol for 15 min. Then, the wells were decanted, air dried, and stained with 200 μL of 2% CV for 15 min. Excess stain was rinsed off gently by water, the plates were air dried, and the bound dye was solubilized with 200 μL of 33% glacial acetic acid. The OD was measured using spectrophotometer and used to calculate the strength of biofilm [51].

The ability of disinfectant solution to inhibit biofilm formation on soft CL (Clear vision, Korea) was also tested using the same procedure with some modifications [53]. Pieces of soft CL were placed in each well; 200 μL of ethanol-acetone (80:20) was used for solubilization of biofilm. The optical density was determined at 630 nm. Each experiment was performed in three replicates.

### Effectiveness of screened compounds on removal of preformed biofilm

Overnight cultures of isolates from TSA plates were inoculated in TSB. The turbidity was adjusted to 0.5 McFarland and then diluted to 1:100. Aliquots of 100 μL were distributed in 96-well microtitre plate containing 100 μL TSB-glu and incubated for 48 hours at 37 °C. The content of each well was aspirated, washed three times with sterile PBS; 200 μL of two fold serial dilutions of the tested compounds (starting from 8, 4 and 2 fold MIC) were added to each well then incubated for 24 hrs at 37 °C. The wells were decanted and washed three times with sterile PBS, fixed with 200 μL of 99% methanol, air dried, and stained for 15 min with 200 μL of 2% Hucker CV. Excess stain was rinsed by water, plates were air dried, dye was solubilized with 200 μL of 33% glacial acetic acid and OD of de-staining solution was measured using spectrophotometer [51].

For CL, The experiment was repeated with the same procedure with addition of pieces of CL in each well, and 200 μL of ethanol-acetone (80:20) was used for solubilisation of biofilm. The optical density was determined at 630 nm. Each experiment was performed in three replicates [53].

## Abbreviations

Cl: Contact lens
CV: Crystal Violet
MIC: minimum inhibitory concentration
OD: Optical Density
